# Organic Acids Improve Growth Performance with Potential Regulation of Redox Homeostasis, Immunity, and Microflora in Intestines of Weaned Piglets

**DOI:** 10.3390/antiox10111665

**Published:** 2021-10-22

**Authors:** Xin-Dong Xiang, Zhang-Chao Deng, You-Wei Wang, Hua Sun, Li Wang, Yan-Ming Han, Yuan-Yuan Wu, Jian-Gao Liu, Lv-Hui Sun

**Affiliations:** 1 Hubei Hongshan Laboratory, College of Animal Science and Technology, Huazhong Agricultural University, Wuhan 430070, China; xiangxindong.hzau.edu.cn@webmail.hzau.edu.cn (X.-D.X.); dengzc@webmail.hzau.edu.cn (Z.-C.D.); huasun@webmail.hzau.edu.cn (H.S.); 2 Hubei Key Laboratory of Embryonic Stem Cell Research, School of Basic Medicine Science, Hubei University of Medicine, Shiyan 442000, China; 6wyw6@163.com; 3 Institute of Animal Science, Guangdong Academy of Agricultural Sciences, Guangzhou 510640, China; 4 Ministry of Agriculture Key Laboratory of Animal Nutrition and Feed Science in South China, Guangzhou 510640, China; 5 Trouw Nutrition, 773811 Amersfoort, The Netherlands; Yanming.Han@trouwnutrition.com (Y.-M.H.); Yuanyuan.Wu@trouwnutrition.com (Y.-Y.W.); 6 Guangzhou Liuhe Feed Company Limited, Guangzhou 511400, China; 7 Key Laboratory of Feed and Livestock and Poultry Products Quality & Safety Control, Ministry of Agriculture, Chengdu 610110, China

**Keywords:** weanling pigs, acidifiers, performance, redox status, immunity, gut microbiota

## Abstract

The objective of this study is to evaluate the effects of organic acids on piglet growth performance and health status. A total of 360 weanling pigs (5.3 ± 0.6 kg) were randomly allotted to 3 treatment groups with 12 replicates of 10 pigs/pen. Piglets were fed the same basal diet and given either water (control) or water plus 2.0 L/Ton organic acid (OA) blends, such as OA1 or OA2, respectively, for 7 weeks. Compared to the control, OA1 and OA2 improved growth performance and/or reduced the piglets’ diarrhea rate during the various periods and improved small intestinal morphology at days 14 and/or 49. OA1 and OA2 also increased serum CAT and SOD activities and/or T-AOC and, as expected, decreased MDA concentration. Moreover, at day 14 and/or day 49, OA1 and OA2 increased the jejunal mRNA levels of host defense peptides (*PBD1*, *PBD2*, *NPG1,* and *NPG3*) and tight junction genes (claudin-1) and decreased that of cytokines (*IL-1β* and *IL-2*). Additionally, the two acidifiers regulated the abundance of several cecum bacterial genera, including *Blautia*, *Bulleidia*, *Coprococcus*, *Dorea*, *Eubacterium*, *Subdoligranulum,* and *YRC2*. In conclusion, both of the organic acid blends improved piglet growth performance and health status, potentially by regulating intestinal redox homeostasis, immunity, and microflora.

## 1. Introduction

Early weanling piglets are exposed to a variety of stress factors, such as psychological, nutritional, and environmental stress [1,2,3], resulting in induced growth retardation, diarrhea, morbidity, and mortality [4,5]. These metabolic disorders, induced by weanling stress, are related to the impairment of inflammatory response, redox balance, intestinal health, and microbiota homeostasis [6,7,8]. Therefore, it is of great significance to develop effective strategies for the improvement of the piglets’ health during the weaning period.

Organic acids have long been recognized as one of the alternative choices to replace antibiotics in diets for weanling pigs [9]. It can reduce diarrhea and improve the nutrient digestibility and growth performance of piglets, which are mainly related to improving gastrointestinal tract health, including decreasing digesta pH values and pathogenic bacteria and maintaining the balance of microflora [10,11,12]. Specifically, formic acid and its salts have an antimicrobial ability and can reduce digesta pH, thus improving digestive enzyme activities [12]. As preservative and antifungal agents, acetic and propionic acids play important roles, mainly as antibacterial agents and against molds, respectively [13]. These short-chain fatty acids also play important roles in the regulation of the immune system and microbiota homeostasis [14,15,16]. Lactic acid is also widely applied to inhibit the growth of key microbial pathogens [13]. Sorbic acid is a long chain unsaturated fatty acid that has a strong antimicrobial activity by suppressing the activities of key enzymes and transport systems and inducing a proton flux into the cells of bacteria [17]. Citric acid is a well-studied organic acid in weanling pigs that has an antibacterial capacity and can act as an energy supply via entering the tricarboxylic acid cycle [9]. 

Since various organic acids play different roles in maintaining the health status of gastrointestinal tract health, the two organic acid blend products were developed based on their unique advantages. The objective of the current study is to evaluate the effects of the two acidifier products, organic acid 1 (OA1) and organic acid 1 (OA2), on weanling piglet growth performance, redox homeostasis, and intestinal health status. 

## 2. Materials and Methods

### 2.1. Piglets, Dietary Treatments, and Sample Collections

The animal protocol was approved by the Institutional Animal Care and Use Committee of Huazhong Agricultural University, China. A total of 360 eighteen-day-old Pig Improvement Company (PIC) weanling pigs (5.3 ± 0.6 kg) were randomly allotted into 3 dietary treatment groups with 12 replicates of 10 pigs/pen (5 barrows and 5 gilts). A basal diet with typical commercial ingredients was prepared (Table S1). The two commercial organic acids blends were produced by Nutreco N.V. (Amsterdam, Netherlands). The piglets from the three groups were given drinking water (Control) or drinking water with added OA1 (mainly containing formic acid (31.0%), ammonium formate (23.0%), and acetic acid (8.3%), with water as a solvent) or OA2 (mainly containing formic acid (13.0%), ammonium formate (19.0%), acetic acid (10.0%), propionic acid (13.0%), sorbic acid (0.5%), lactic acid (1.0%), and citric acid (0.5%), with water as a solvent) at 2.0 L/Ton. The final pH of the water was 8.17, 4.21, and 4.88 in the groups of Control, OA1, and OA2, respectively. Feed and water were given ad libitum during the experimental period. A three-phase feeding program was followed, with weanling 1–14 days post-weanling, 15–28 days post-weanling, 29–49 days post-weanling as Phase 1, Phase 2, and Phase 3, respectively. Feed intake, diarrhea, and mortality were recorded daily, and body weight was measured at day 14 post-weanling and day 49 post-weanling. On the last day of Phase 1 and Phase 3, 6 male piglets per treatment, at average body weight from different replicates, were selected and slaughtered. Blood, small intestinal, and cecum digesta samples were collected for biochemical, histologic examination, and microbial analyses [18,19]. Briefly, the serum was prepared by centrifugation at 4 °C at 1000× *g* for 15 min and stored at −80 °C before use. The small intestinal segments were perfused with ice-cold isotonic saline, minced with surgical scissors, divided into aliquots, and stored at −80 °C until analysis. 

### 2.2. Redox Status, Digestive Enzyme Activity, and Histologic Analysis

Total antioxidant capacity (T-AOC), glutathione peroxidase (GPX), catalase (CAT) and superoxide dismutase (SOD) activities and malondialdehyde (MDA) and protein carbonyl (PC) concentrations were determined by their specific assay kits (A015-1-2, A005-1-2, A007-1-1, A001-3-2, A003-1-2, and A087-1-2) from the Nanjing Jiancheng Bioengineering Institute of China. Protein concentration was analyzed by the bicinchoninic acid assay [19]. Jejunal chyme lipase, α-amylase, and neutral protease activities were measured by their specific assay kits (A054-1, C016-1, A080-3-1; Nanjing Jiancheng Bioengineering Institute, Nanjing, China) [20]. Small intestinal histopathology and morphology were analyzed as previously described [18]. Briefly, the duodenum, jejunum, and ileum tissues were washed with saline, fixed in 10% neutral buffered formalin, embedded in paraffin, sectioned at 5 μm, and then stained with hematoxylin and eosin. All slides were examined by a microscope. 

### 2.3. Real-Time q-PCR Analyses

Real-time q-PCR analyses of the jejunum samples were carried out as previously described [18]. Briefly, total RNA was isolated from the jejunum of 6 piglets from each group, and the RNA samples were quantified and qualified by a NanoDrop spectrophotometer. The mRNA levels of cytokines genes (*Interleukin* (*IL*)*-1β*, *IL-2* and *IL-6*), host defense peptide genes (*porcine β-defensin* (*PBD*) *1*, *PBD2*, *epididymis protein 2 splicing variant C* (*PEP2C*), *proline/arginine-rich peptide of 39 amino acids* (*PR39*), *protegrin* (*NPG*) *1* and *NPG3*) and tight junction genes (*claudin-1*, *claudin-2*, *occludin*, *zonula occludens-1* (*ZO-1*), and *myosin light chain kinase* (*MLCK*)) were measured by qPCR (CFX384; Bio-Rad). The primer information of these assayed genes and the housekeeping gene *glyceraldehyde-3-phosphate dehydrogenase* (*GAPDH*) [21,22] are presented in Table S2. The 2^−ddCt^ method was used for quantification, with *GAPDH* as a reference gene [18]. 

### 2.4. Pyrosequencing of Bacterial 16S rRNA Gene Amplification

Microbiota analyses of the cecum digesta samples were conducted as previously described [23]. Briefly, microbial genomic DNA was extracted from cecum digesta using the DNA stool mini kit (Tiangen, Beijing, China), according to the instructions of the manufacturer; a Nanodrop spectrophotometer was used to estimate DNA quantity and quality. The V3–V4 region of the bacterial 16S ribosomal RNA gene was amplified with the primers 338F (5′-ACTCCTACGGGAGGCAGCA-3′) and 806R (5′-GGACTACHVGGGTWVTAAT-3′) by PCR. The PCR products were separated by electrophoresis with a 1.5% agarose gel and separated and purified using the QIAquick Gel Extraction Kit (Qiagen, Germany). The purified products were sequenced using the Illumina Miseq platform (Personal Biotechnology Co., Ltd., Shanghai, China). The alpha diversity index (Chao1 index and observed OTUs), Jaccard distances for principal coordinate analysis (PCoA), and LDA effect Size (LEfSe) analysis were generated or calculated by personal genescloud (https://www.genescloud.cn/home, accessed on 15 September 2021).

### 2.5. Statistical Analyses

Statistical analyses of microbial data were performed using GraphPad Prism (San Diego, CA, USA), and *p*-values were tested by Wilcoxon’s rank-sum test. Data visualization was performed using R V.3.5.0 using the ggplot2 and corrplot packages. Other than that, statistical analyses were performed using SPSS version 13 (Chicago, IL, USA). Data are presented as mean ± SD. Levene’s test of homogeneity of variance was used to evaluate the unequal variances before ANOVA analysis. Data were analyzed by one-way ANOVA, with a significance level of *p* < 0.05, and tendency was set at 0.05 ≤ *p* < 0.10; the Tukey-Kramer method was used for multiple mean comparisons. Welch’s ANOVA was used in the case of unequal variances, followed by Dunnett’s T3 test for post hoc comparisons.

## 3. Results

### 3.1. Growth Performance

The performance results are presented in Table 1. Compared to the control, OA1 significantly increased or tended to increase (*p* < 0.05 or 0.10) feed intake (11.0%) and body weight gain (13.1%) during days 1–14 and body weight gain (4.3%) during days 1–49. Although OA2 did not affect (*p* > 0.10) the feed intake and body weight gain throughout the experiment, it significantly reduced or tended to reduce (*p* < 0.05 or 0.10) the feed/gain ratio (2.1%) during days 15–49 and the diarrhea rate (31.4–53.5%) of piglets throughout the study compared to control. Interestingly, OA1 has worse results (*p* < 0.05) on the diarrhea rate (37.1–96.6%), but it significantly had or tended to have better results (*p* < 0.05 or 0.10) on feed intake (5.6–10.8%) and body weight gain (5.5–12.4%) than OA2 throughout the experiment. No difference (*p* > 0.10) was found in the mortality rate among the three groups throughout the study.

### 3.2. Serum Redox Status 

The serum redox status results are presented in Table 2. Compared to the control, OA1 tended to increase (*p* < 0.10) CAT activity (25.6%), while OA2 tended to increase (*p* < 0.10) T-AOC activity (30.4%) in the serum of piglets at day 14. Interestingly, OA1 increased (*p* < 0.05) SOD activity by 13.5% and OA2 significantly increased or tended to increase (*p* < 0.05 or 0.10) both SOD and T-AOC activities in serum by 11.6–14.0%, respectively, compared to control at day 49. Notably, compared to control, both OA1 and OA2 reduced (*p* < 0.05) MDA concentrations by 35.7–45.7%, respectively, in the serum of piglets at day 49.

### 3.3. Small Intestinal Histology and Chyme Digestive Enzyme Activity

**Figure 1:**
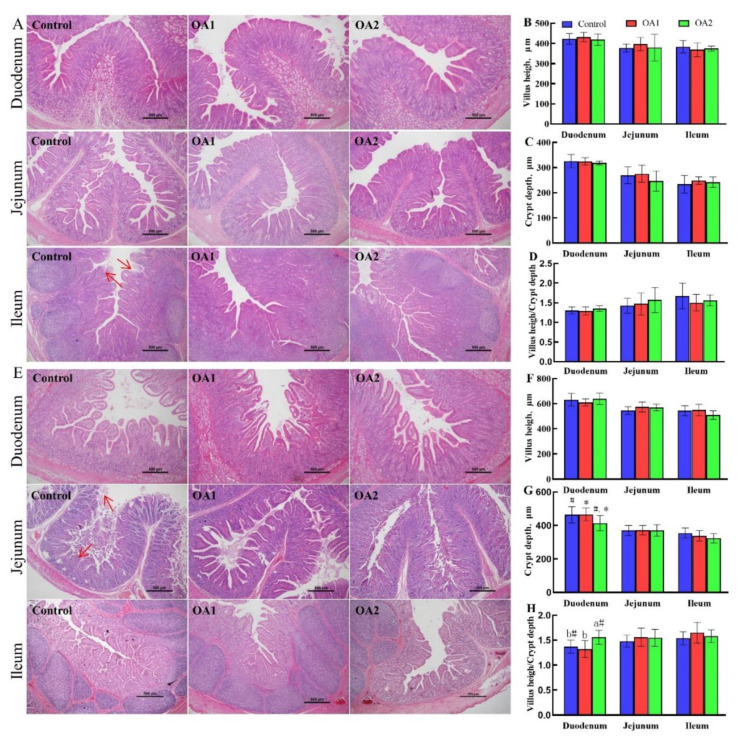
Effects of acidifiers on the gut morphology of the small intestine of piglets. The histopathology of the duodenum, jejunum, and ileum at day 14 (**A**) and day 49 (**E**). The morphology of villus height (**B**), crypt depth (**C**), and villus height/crypt depth (**D**) at day 14. The morphology of villus height (**G**), crypt depth (**F**), and villus height/crypt depth (**H**) at day 49. Values are means ± SD, *n* = 6. Labeled means in a row with different letters differ, *p* < 0.05. A given 2 means labeled with ^#^ indicates a tendency, 0.05 ≤ *p* < 0.10. At the red arrow, the intestinal villi were broken off and the intestinal epithelial cells were denatured and necrotic.

The small intestinal histology results are presented in Figure 1. The histopathology results showed that fragmentation of villi and slight vacuolar degeneration of epithelial cells were observed in the ileum (day 14) and jejunum (day 49) of the control group (Figure 1A,D, red arrow). Notably, the intestinal morphology results showed that OA2 tended to reduce (*p* < 0.10) crypt depth (Figure 1G) and significantly increased or tended to increase (*p* < 0.05 or 0.10) the ratio of villus height:crypt depth (Figure 1H) in the duodenum at day 49 compared to those of the control and OA1. The chyme digestive enzyme activity results are presented in Table 3. The jejunal chyme α-amylase, lipase, and neutral protease activities were not affected (*p* > 0.10) by both OA1 and OA2 at both days 14 and 49.

### 3.4. Expression of Cytokine-, Tight Junction-, and Host Defense Peptide-Related Genes

**Figure 2:**
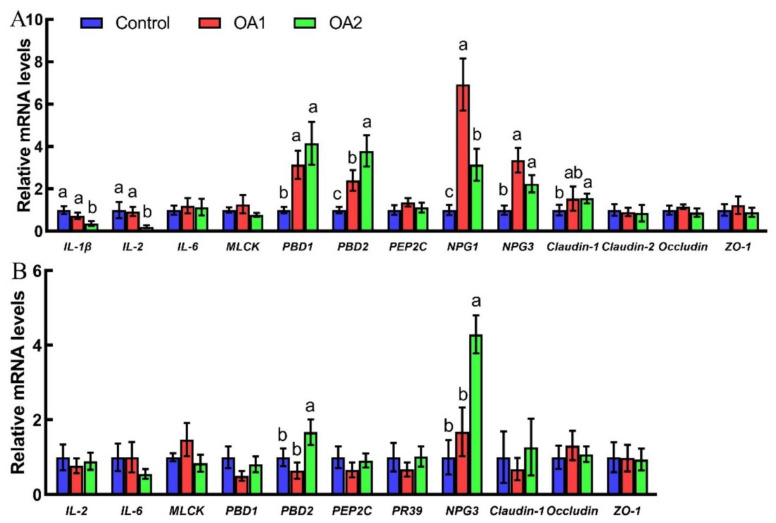
Effects of acidifiers on relative mRNA abundance of cytokines, tight junction genes, and defense peptides in the jejunum of piglets at day 14 (**A**) and day 49 (**B**). Values are means ± SD, *n* = 6. Labeled means without a common letter differ, *p* < 0.05.

The effects of acidifiers on gene expression results are shown in Figure 2. Compared to the control, both OA1 and OA2 increased (*p* < 0.05) host defense peptides (*PBD1*, *PBD2*, *NPG1*, and *NPG3*), while only OA2 decreased (*p* < 0.05) cytokines (IL-1β and IL-2) and increased (*p* < 0.05) tight junction genes (*claudin-1*) at the mRNA level in the jejunum at day 14. Notably, OA2 decreased (*p* < 0.05) *IL-1β*, *IL-2,* and *NPG1*, while it increased (*p* < 0.05) *PBD2* at the mRNA level in the jejunum compared to OA1 at day 14. At day 49, OA2 increased (*p* < 0.05) the mRNA abundance of *PBD2* and *NPG3* in the jejunum compared to those of the control and OA1.

### 3.5. Gut Microbiota

**Figure 3:**
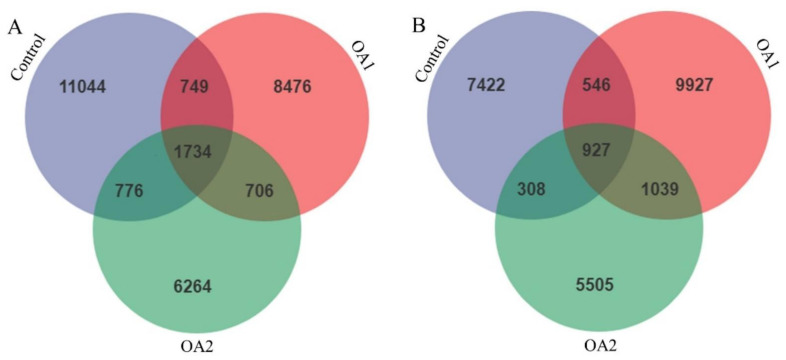
Number of identified ASVs in various comparisons in cecum chyme at day 14 (**A**) and day 49 (**B**). Each ellipse represents a treatment; the numbers in the diagrams represent how many ASVs are unique in the three groups and how many are shared as the areas intersect. ASVs, amplicon sequence variants.

**Figure 4:**
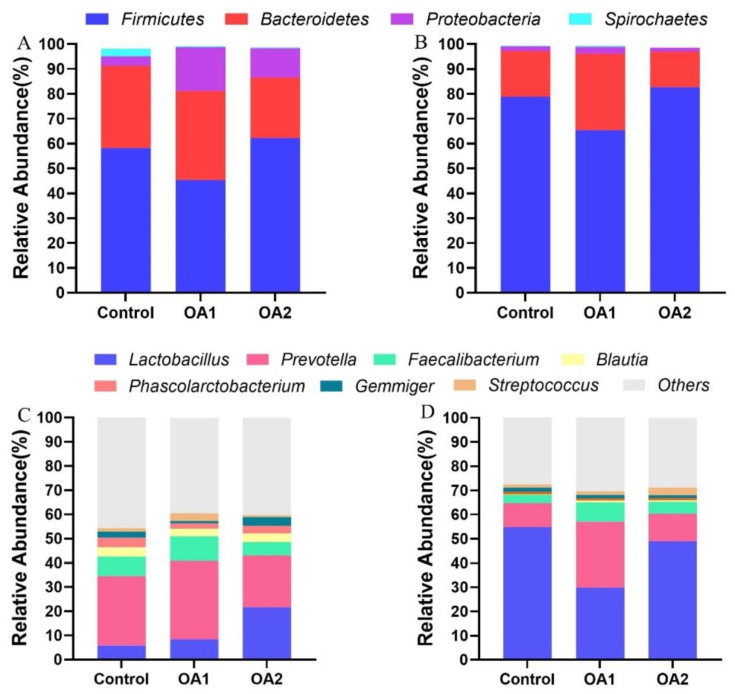
The analysis of composition of bacteria in cecum chyme. The phylum level of composition of bacteria at day 14 (**A**) and day 49 (**B**). The genus level of composition of bacteria at day 14 (**C**) and day 49 (**D**). The color-coded bar plot shows the relative abundance of bacterial phyla across the different groups.

**Figure 5:**
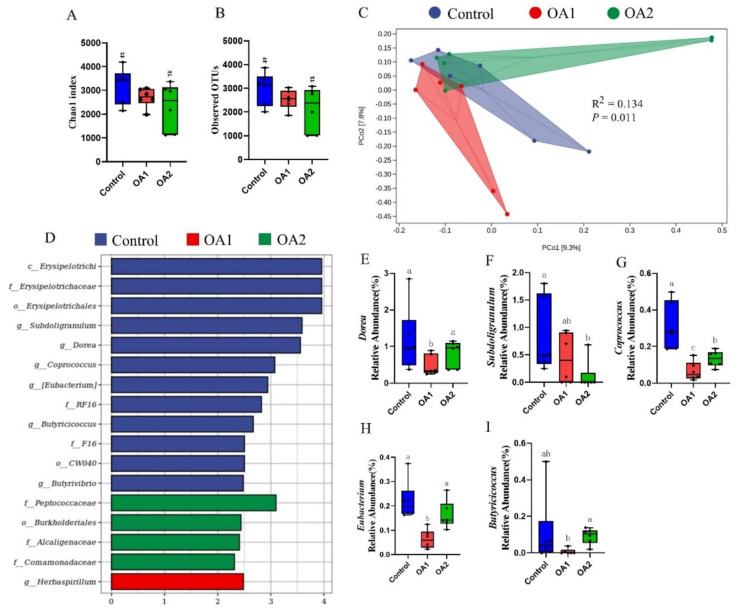
Gut microbiota analysis of cecum chyme at day 14. Comparison of alpha diversity index (Chao1 index and observed OTUs) among the three groups (**A**,**B**). Principal coordinates analysis (PCoA) based on Jaccard distances (**C**). Each point in the figure represents a sample, and different colored points indicate different groups. *p*-values were tested by permutational multivariate analysis of variance (Adonis). LEfSe analysis effect size identified the most differentially abundant taxa in the cecal chyme microbiota community of the three groups, and only taxonomies of LDA score >2 are shown (**D**). Relative abundances of *Dorea*, *Subdoligranulum*, *Coprococcus*, *Eubacterium*, and *Butyricicoccus* among the three groups (**E**–**I**). Labeled means with different superscript letters are significantly different (*p* ≤ 0.05) and with ^#^ indicates a tendency (0.05 ≤ *p* < 0.10) by Wilcoxon’s rank-sum test (*n* = 6). LDA, linear discriminant analysis.

**Figure 6:**
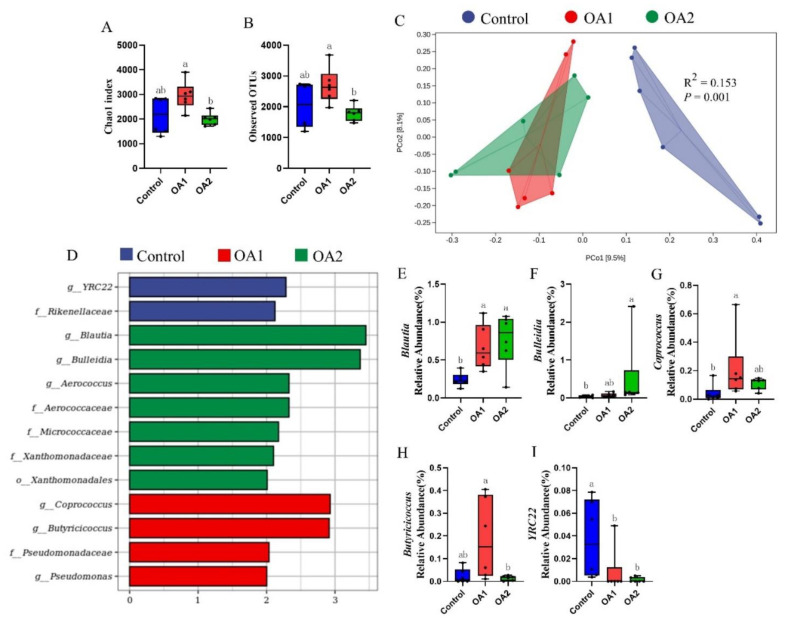
Gut microbiota analysis of cecum chyme at day 49. Comparison of alpha diversity index (Chao1 index and observed OTUs) among three groups (**A**,**B**). Principal coordinates analysis (PCoA) based on Jaccard distances (**C**). Each point in the figure represents a sample, and different colored points indicate different groups. *p*-values were tested by permutational multivariate analysis of variance (Adonis). LEfSe analysis effect size identified the most differentially abundant taxa in the cecal chyme microbiota community of the three groups, and only taxonomies of LDA score >2 are shown (**D**). Relative abundances of *Blautia*, *Bulleidia*, *Coprococcus*, *Butyricicoccus*, and *YRC22* among the three groups (**E**–**I**). Labeled means with different superscript letters are significantly different (*p* ≤ 0.05) by Wilcoxon’s rank-sum test (*n* = 6). LDA, linear discriminant analysis.

The effects of acidifiers on gut microbiota are shown in Figure 3, Figure 4, Figure 5 and Figure 6. The qualified sequences were clustered into amplicon sequence variants (ASVs) according to sequence similarity (>97%; File S1). Interestingly, more ASVs were identified in the three groups, and the three diet groups shared 1734 ASVs at day 14 (Figure 3A) and 927 ASVs at day 49 (Figure 3B) in cecum chyme. From the analysis of taxonomic composition, both OA1 and OA2 altered the relative abundance of intestinal bacteria at both the phylum and genus levels. All the qualified sequences from the cecum chyme samples were then assigned to 25 known phyla (File S2). Of them, four phyla, *Firmicutes*, *Bacteroidetes*, *Proteobacteria*, and *Spirochaetes,* were predominantly found (Figure 4A,B). Moreover, 50 predominant genera were assigned from all the qualified sequences (File S3), and seven genera, including *Lactobacillus*, *Prevotella*, *Faecalibacterium*, *Blautia*, *Phascolarctobacterium*, *Gemmiger*, and *Streptococcus,* were predominantly found (Figure 4C,D).

At day 14, the alpha diversity index (Chao1 index and observed OTUs) for the microbiome analysis of OA2 tended to decrease (*p* < 0.10) when compared to the control (Figure 5A,B). Interestingly, the alpha diversity index of OA1 was higher (*p* < 0.05) than of OA2 (Figure 6A,B) at day 49. The results of principal coordinates analysis using Jaccard distances showed that the control was differentiated (*p* = 0.011) at day 14 (Figure 5C) and was greatly (*p* = 0.001) differentiated at day 49 (Figure 6C) compared to those of OA1 or OA2.

To identify which bacterial taxa were distinctly different among the three groups, LEfSe analysis with LDA scores >2 was used. The results identified 12 discriminative features in the microbiota of the control, 1 in OA1, and 4 in OA2 at day 14 (Figure 5D) and identified 2 in the control, 4 in OA1, and 7 in OA2 at day 49 (Figure 6D). Moreover, at day 14, the abundance comparisons of predominant genera revealed that *Dorea*, *Coprococcus*, and *Eubacterium* in OA1 were significantly reduced (*p* < 0.05), whereas *Subdoligranulum* and *Coprococcus* in OA2 were greatly decreased (*p* < 0.05) when compared to the control (Figure 5E–I). However, at day 49, *Coprococcus* and *Blautia* in OA1 and *Blautia* and *Bulleidia* in OA2 were significantly increased (*p* < 0.05), whereas *YRC22* in OA1 and OA2 were greatly decreased (*p* < 0.05) when compared to the control (Figure 6E–I).

## 4. Discussion

The current study shows that drinking water supplementation of organic acids can improve the performance of weanling pigs. Specifically, OA1 significantly increased or tended to increase feed intake and body weight gain of piglets during days 1–14 and 1–49, while OA2 significantly reduced or tended to reduce the feed/gain ratio during days 15–49 and the diarrhea rate throughout the study. These outcomes are similar to previous studies, which have reported that organic acids alone or in combination can improve the performance of weanling pigs [7,11,24,25], chickens [26,27,28], and rabbits [29]. The beneficial effects of these organic acids on the performance of animals have been associated with their ability to reduce digesta pH value, thus improving the antibiotics’ roles and digestive enzyme activities in the gastrointestinal tract [7,11,24,25,26,27,28,29]. Notably, OA1 displayed better ability in the improvement of feed intake and body weight gain, while OA2 exerted a better ability to increase feed conversion efficiency and decrease the diarrhea rate. These discrepancies could be due to the different composition of the two acidifiers and their acidification capacity of OA1 (pH 4.21) and OA2 (pH 4.88) to the drinking water [30]. 

Redox imbalance is usually triggered by early weanling stress, resulting in organ damage and disease susceptibility in weanling piglets [3,31,32,33,34]. Interestingly, both of the two organic acid blends significantly improved or tended to improve the redox status, including the enhancement of the serum T-AOC, SOD, and/or CAT activities of piglets. This may be owed to the piglets ingesting acidifiers containing formic acid, acetic acid, propionic acid, butyric acid, lactic acid, citric acid and/or sorbic acid, which have a function in maintaining redox homeostasis in animals [35,36,37]. 

The gastrointestinal tract is particularly susceptible to weanling stress, which can impair gastrointestinal morphology, permeability, and function [38]. Notably, both of the acidifiers prevented the fragmentation of villi, and slight vacuolar degeneration of epithelial cells in the ileum (day 14) and jejunum (day 49) were observed in the control group. Meanwhile, OA2 significantly reduced or tended to reduce crypt depth and increased the ratio of villus height:crypt depth in the duodenum at day 49 compared to those of the control and OA1. These outcomes are in agreement with previous studies, which showed organic acids can improve intestinal morphology [39]. Although digestive enzyme activities were not affected by the two organic acid blends, they were reduced at day 49 compared to those on day 14. This might be due to the piglets ingesting more feed; thus, these digestive enzyme activities were diluted by chyme from the jejunum [40]. Moreover, the two acidifiers, OA1 and/or OA2, decreased pro-inflammatory cytokines (*IL-1β* and *IL-2*) [41,42] and increased host defense peptides (*PBD1*, *PBD2*, *NPG1*, and *NPG3*) [43,44] and tight junction (*claudin-1*) gene expression at the mRNA level in the jejunum at day 14 and 49 compared to those of the control. Notably, OA2 decreased *IL-1β* and *IL-2* while increasing *PBD2* and *NPG3* at the mRNA level in the jejunum compared to OA1 at days 14 and/or 49. These results could explain why the two acidifiers have better intestinal morphology than those of the control and why the OA2 group is better than the OA1 group. The chyme digestive enzyme activities were not affected by the two acidifiers, which was inconsistent with the previous report [45]. This discrepancy might be due to the different animal species, physiological stages, dietary structure, and composition of acidifiers [38,46]. 

Since gut microbes also play an active role in maintaining the intestinal health of weanling pigs [38,47,48], 16S rRNA gene sequencing technology was used to analyze and compare the cecal microbiota composition of the weaned pigs. Compared with the control, the abundance of predominant genera of *Blautia* was increased and YRC22 was decreased in both OA1 and OA2, while *Dorea* and *Eubacterium* in OA1 and *Subdoligranulum* in OA2 were decreased and *Bulleidia* in OA2 was increased at days 14 and/or 49. *Blautia* plays the roles of an antibacterial agent and alleviating inflammatory disease characteristics [49], and *Dorea* can metabolize mucin and induce pro-inflammatory cytokines [50]; thus, increasing *Blautia* and decreasing *Dorea* by OA1 and/or OA2 may improve the immunity status of the gut of piglets. *Eubacterium* and *Subdoligranulum* can produce butyrate, which plays a critical role in energy homeostasis and immunomodulation in the gut [51,52]. Strikingly, these genera were decreased in OA1 or OA2, which might be due to an adaptation mechanism where the piglets have already ingested adequate organic acids with similar functions from their drinking water, thus suppressing the growth of these genera [12,53]. Since *Bulleidia* is a hyperglycemia-associated bacterial genus [54] and the function of *YRC22* is unknown [55], the effects of changes of these bacteria by OA1 and OA2 are unknown. Strikingly, *Coprococcus* can also produce butyrate [56], but it is hard for us to explain why *Coprococcus* was decreased in both OA1 and OA2 at day 14, while it was increased in OA2 at day 49 compared to those of the control. Therefore, its role in organic-acid-mediated gastrointestinal health needs to be further explored. 

## 5. Conclusions

In summary, the current study has illustrated that drinking water supplementation of both organic acid blends can improve the performance and health status of weanling pigs, as further evidenced by improvement of redox homeostasis and gastrointestinal morphology. The protective mechanisms of these two organic acids against weanling stress in piglets are associated with (1) the regulation of cytokines and host defense peptides and tight junction gene expression in the jejunum; and (2) maintaining the balance of microflora in the intestine.

## Figures and Tables

**Table 1:** Effects of organic acids on growth performance and diarrhea rate ^1^.

	Control	OA1	OA2	*p*-Value
Days 1–14				
Feed intake, kg/piglet	2.92 ± 0.25 ^b^	3.24 ± 0.45 ^a^	2.89 ± 0.24 ^b^	0.02
Body weight gain, kg/piglet	1.68 ± 0.17 ^b^	1.90 ± 0.24 ^a^	1.69 ± 0.16 ^b^	0.02
Feed/gain ratio, kg/kg	1.75 ± 0.16	1.71 ± 0.18	1.72 ± 0.24	0.88
Diarrhea rate, %	10.2 ± 3.1 ^a^	9.6 ± 2.9 ^a^	7.0 ± 2.7 ^b^	0.03
Mortality rate, %	0.83 ± 2.89	0.00 ± 0.00	1.67 ± 3.89	0.36
Days 15–49				
Feed intake, kg/piglet	24.0 ± 1.3 ^a,b^	24.4 ± 1.7 ^a^	23.1 ± 1.3 ^b^	0.08
Body weight gain, kg/piglet	16.7 ± 0.8	17.3 ± 1.2 ^#^	16.4 ± 1.0 ^#^	0.13
Feed/gain ratio, kg/kg	1.43 ± 0.05 ^#^	1.41 ± 0.05	1.40 ± 0.03 ^#^	0.23
Diarrhea rate, %	1.27 ± 0.73 ^a^	1.16 ± 0.81 ^a^	0.59 ± 0.54 ^b^	0.05
Mortality rate, %	0.83 ± 2.89	3.33 ± 4.92	2.50 ± 4.52	0.35
Days 1–49				
Feed intake, kg/piglet	26.9 ± 1.5 ^a,b^	27.7 ± 1.9 ^a^	26.0 ± 1.4 ^b^	0.05
Body weight gain, kg/piglet	18.4 ± 0.9 ^b,#^	19.2 ± 1.3 ^a,#^	18.1 ± 1.1 ^b^	0.06
Feed/gain ratio, kg/kg	1.46 ± 0.05	1.44 ± 0.05	1.43 ± 0.04	0.26
Diarrhea rate, %	4.01 ± 1.03 ^a^	3.80 ±1.32 ^a^	2.58 ± 0.98 ^b^	0.01
Mortality rate, %	1.67 ± 3.89	3.33 ± 4.92	4.17 ± 6.69	0.51

^1^ Values are means ± SD, *n* = 12. Labeled means in a row with different letters differ, *p* < 0.05. A given 2 means labeled with ^#^ indicates a tendency, 0.05 ≤ *p* < 0.10.

**Table 2:** Effects of organic acids on the serum antioxidant indexes ^1^.

	Control	OA1	OA2	*p* value
Day 14				
T-AOC, U/L	125 ± 24 ^b^	150 ± 36 ^a,b^	163 ± 30.4 ^a^	0.13
GPX, U/mL	635 ± 57	594 ± 49	664 ± 123	0.36
SOD, U/mL	37.6 ± 4.0	39.7 ± 4.0	40.7 ± 4.7	0.45
CAT, U/mL	3.9 ± 1.2 ^#^	4.9 ± 0.3 ^#^	5.2 ± 2.4	0.35
MDA, nmol/mL	2.3 ± 0.9	2.7 ± 0.7	4.1 ± 2.1	0.13
PC, nmol/mL	1.3 ± 0.8	0.9 ± 0.4	1.2 ± 0.4	0.67
Day 49				
T-AOC, U/L	189 ± 22 ^#^	197 ± 26	211 ± 19 ^#^	0.27
GPX, U/mL	889 ± 89	908 ± 45	855 ± 57	0.40
SOD, U/mL	41.4 ± 1.0 ^b^	47.0 ± 1.6 ^a^	47.2 ± 2.1 ^a^	0.00
CAT, U/mL	2.5 ± 0.6	2.7 ± 1.0	2.6 ± 0.9	0.93
MDA, nmol/mL	7.0 ± 1.1 ^a^	3.8 ± 1.8 ^b^	4.5 ± 1.5 ^b^	0.01
PC, nmol/mL	0.5 ± 0.2	0.5 ± 0.2	0.4 ± 0.2	0.80

^1^ Values are means ± SD, *n* = 6. Labeled means in a row with different letters differ, *p* < 0.05. A given 2 means labeled with ^#^ indicates a tendency, 0.05 ≤ *p* < 0.10. T-AOC, total antioxidative capacity; GPX, glutathione peroxidase; CAT, catalase; SOD, superoxide dismutase; MDA, malondialdehyde; PC, protein carbonyl.

**Table 3:** Effects of organic acids on the digestive enzyme activity of jejunum chime ^1^.

	Control	OA1	OA2	*p*-Value
Day 14				
α-amylase, U/g	69.2 ± 27.4	88.7 ± 22.2	81.4 ± 26.7	0.47
Lipase, U/g	57.9 ± 23.8	66.7 ± 23.0	43.7 ± 22.8	0.26
Neutral protease, U/g	61.8 ± 16.7	73.5 ± 13.8	62.0 ± 18.2	0.39
Day 49				
α-amylase, U/g	27.0 ± 12.4	22.7 ± 8.1	25.5 ± 15.0	0.82
Lipase, U/g	46.3 ± 19.6	31.7 ± 12.0	30.8 ± 5.8	0.15
Neutral protease, U/g	46.2 ± 15.0	48.8 ± 15.3	40.3 ± 18.0	0.87

^1^ Values are means ± SD, *n* = 6. Labeled means in a row without a common letter differ, *p* < 0.05.

## Data Availability

Data are contained within the article.

## Supplementary Materials

The following are available online at https://www.mdpi.com/article/10.3390/antiox10111665/s1. Table S1: Basal diet composition and nutrient level; Table S2: List of primers used for Q-PCR analysis; File S1: Amplicon sequence variants; File S2: Phylum level composition of bacteria; File S3: Genus level composition of bacteria.
