# Supplementary material for: Organic Acids Improve Growth Performance with Potential Regulation of Redox Homeostasis, Immunity, and Microflora in Intestines of Weaned Piglets
Source: Antioxidants (Basel). 2021 Oct 22;10(11):1665. doi: 10.3390/antiox10111665 (PMC8615128; doi:10.3390/antiox10111665)
Supplement: Supplementary file 1 [file antioxidants-10-01665-s001.zip › Supplements/Supplemental Tables.pdf]

Table S1. Basal diet composition and nutrient level

Ingredients	Phase 1 (Weanling-14 days post weanling)	Phase 2 (15-28 days post weanling)	Phase 3 (29-49 days post weanling)
CORN	44.20	55.31	62.57
SBM46%	10.14	15.50	18.91
Wheat flour	5.00	8.00	8.00
Fishmeal	3.00	-	-
Brewer's yeast distillers (60%CP)	2.00	-	-
Whey powder	20.00	5.00	-
Extruded soybean	6.00	5.00	5.00
Fermented soybean meal	4.00	4.00	-
Sucrose	2.00	2.00	-
Soybean oil	0.89	1.04	1.34
Dicalcium phosphate III (21%)	0.43	0.93	0.98
Limestone	0.40	0.85	0.94
L-lysine hydrochloride (98.5%)	0.51	0.61	0.55
NaCl	0.02	0.33	0.41
Mineral premix[1]	0.30	0.30	0.30
Vitamin premix[2]	0.30	0.30	0.30
L-Tryptophan (25%)	0.30	0.29	0.24
DL-methionine (98.5%)	0.29	0.27	0.22
L-Threonine (98.5%)	0.16	0.21	0.18
Choline chloride (60%)	0.05	0.05	0.05
Phytase (heat-stable, 20000 FTU/g)	0.01	0.01	0.01
Total	100	100	100
Nutrients level [3]			
CP, %	19.0	18.0	17.5
ME(Mcal/kg)	3.41	3.36	3.36
CF, %	1.82	2.21	2.30
ASH, %	5.04	4.80	4.57
Ca, %	0.60	0.65	0.65
TP, %	0.58	0.54	0.53
aP(P) , %	0.35	0.29	0.27
SID Lys, %	1.35	1.25	1.15
SID Met, %	0.57	0.51	0.45
SID Cys, %	0.24	0.24	0.24
SID Met+Cys, %	0.81	0.75	0.69
SID Thr , %	0.81	0.75	0.69
SID Trp , %	0.24	0.23	0.21
SID Arg, %	0.94	1.01	1.00
SID Ile, %	0.71	0.63	0.59
SID Val, %	0.78	0.70	0.68
SID Leu, %	1.36	1.28	1.27
Phytase (FTU/KG)	1000	1000	1000

[1] Provided the following per kg of diets: Fe, 100 mg; Cu, 20 mg; Mn, 25 mg; Zn, 100 mg; Na2O3, 0.3 mg; I, 0.3 mg.

[2] Provided the following per kg of diets: retinyl acetate, 10000 IU; cholecalciferol 2500 IU; dl-α-tocopheryl acetate, 50 IU; menadione, 5.0 mg; thiamin, 2.0 mg; riboflavin, 5.0 mg; pantothenic acid, 12.0 mg; pyridoxine, 10.0 mg; niacin, 30.0 mg; d-biotin, 0.2 mg; folic acid, 1.5 mg; cyanocobalamin, 0.05 mg; choline chloride 1500 mg.

[3] Nutrient Levels were a calculated value.

Table S2. List of primers used for Q-PCR analysis[1]

Gene	Gene accession number	Forward primer (5′ to 3′)	Reverse primer (5′ to 3′)
IL-1β	NM_214055.1	GCCCAATTCAGGGACCCTAC	GGCGGGTTCAGGTACTATGG
IL-2	NM_213861.1	CCCTTGCACTCATGGCAAAC	TAGCACTCCCTCCAGAGCTT
IL-6	NM_001252429.1	GCAGTCACAGAACGAGTGGA	CTCAGGCTGAACTGCAGGAA
MLCK	XM_001929078.6	ATGGCAATCGGGAGCATGAG	TCCTCCCCGTCTTCTGTTCT
PBD1	NM_214442.2	CCAGCTGGCTGCAGGTATTA	ACTTGGCCTTGCCACTGTAA
PBD2	NM_213738.1	AACCTGCTTACGGGTCTTGG	TTGCCACTGTAACAGGTCCC
PEP2C	BK005522.1	ACCAAGGGTCCCAGAGAACT	GGCTCTGGAGAACAAGCAGT
NPG1	NM_001123149.1	TGACTTCAAGGAGAACGGGC	GGAACCTTGGTGGACCGAAT
NPG3	NM_001123150.1	TGACTTCAAGGAGAACGGGC	ACAATAGCACAGGCCACCTC
PR39	NM_214450.1	TGACTTCAAGGAGAACGGGC	CAAATATGGGGGTCGGGGAC
Claudin-1	NM_001244539.1	GCCACTGTTGGCATGAAGTG	CTGGCATTGACTGGGGTCAT
Claudin-2	NM_001161638.1	ATCCTCTGCTTTTCCTGCCC	CTGTCCACTGGCTCTCACAG
Occludin	NM_001163647.2	CAGGTGCACCCTCCAGATTG	ATGTCGTTGCTGGGTGCATA
ZO-1	XM_021098896.1	GCCAGCTGGAGCTTAGAACA	GGCATCAAGAGGGGCTACTG
GAPDH	NM_001206359.1	GGTCGGAGTGAACGGATTT	ATTTGATGTTGGCGGGAT

[1]*(IL)-1β*, *-2* and *-6*, *Interleukin -1β*, *-2* and *-6*; *MLCK, myosin light chain kinase; PBD1 and 2, Porcine β-Defensin 1* and 2; *PEP2C, epididymis protein 2 splicing variant C, NPG1* and *3*, *protegrin 1* and 3; *PR39, proline/arginine-rich peptide of 39 amino acids*; *ZO-1, zonula occludens-1*; *GAPDH, glyceraldehyde-3-phosphate dehydrogenase*.
